# Genetic Characterization of H5N8 Highly Pathogenic Avian Influenza Viruses Isolated from Falcated Ducks and Environmental Water in Japan in November 2020

**DOI:** 10.3390/pathogens10020171

**Published:** 2021-02-04

**Authors:** Ahmed Magdy Khalil, Yoshikazu Fujimoto, Isshu Kojima, Mana Esaki, Kyonha Ri, Tatsunori Masatani, Tsutomu Matsui, Makoto Ozawa

**Affiliations:** 1 Joint Faculty of Veterinary Medicine, Kagoshima University, Kagoshima 890-0065, Japan; ahmed.magdy.am549@gmail.com (A.M.K.); k7639981@kadai.jp (Y.F.); k0068103@kadai.jp (M.E.); k6459590@kadai.jp (K.R.); masatani@vet.kagoshima-u.ac.jp (T.M.); 2 United Graduate School of Veterinary Science, Yamaguchi University, Yamaguchi 753-8511, Japan; 3 Faculty of Veterinary Medicine, Zagazig University, 44511 Zagazig, Egypt; 4 Joint Graduate School of Veterinary Medicine, Kagoshima University, Kagoshima 890-0065, Japan; k3732120@kadai.jp; 5 Kagoshima Crane Conservation Committee, Izumi, Kagoshima 899-0208, Japan; crane_c@city.izumi.kagoshima.jp

**Keywords:** highly pathogenic avian influenza virus, H5N8, falcated duck

## Abstract

We isolated two highly pathogenic avian influenza viruses (HPAIVs) of subtype H5N8 clade 2.3.4.4b from falcated duck (*Anas falcata*) feces and environmental water collected at an overwintering site in Japan. Our isolates were almost genetically identical to each other and showed high genetic similarity with H5N8 HPAIVs recently isolated in South Korea, a distant part of Japan, and European countries. These results suggest the potential role of falcated ducks in the dissemination of HPAIVs.

Since the detection of A/Goose/Guangdong/1/1996 (H5N1) (Gs/GD96) from domestic poultry in China in 1996, highly pathogenic avian influenza viruses (HPAIVs) of the H5Nx subtype have been circulating in wild and domestic birds [1]. In addition, the hemagglutinin (HA) of the Gs/GD96 strain has evolved into multiple distinct phylogenetic clades, subclades, and lineages worldwide [2]. Since 2014, H5Nx HPAIVs of clade 2.3.4.4 have been circulating in wild and domestic birds in several countries [3,4,5,6], resulting in further classification into four subclades, namely clades 2.3.4.4a–2.3.4.4d [7]. In the winter of 2019/2020, H5N8 HPAIVs belonging to clade 2.3.4.4b caused outbreaks in wild and domestic birds in Europe [8]. Similarly, in the winter of 2020/2021, genetically similar H5N8 HPAIVs from clade 2.3.4.4b were disseminated not only in European countries, but also in South Korea and Japan [9,10], which is most likely due to the migration of wild aquatic birds that are considered natural reservoirs of avian influenza viruses (AIVs) [11], as suggested in previous studies on the dissemination of H5N8 HPAIVs [3,4,5,6]. Here, we describe the isolation of two H5N8 HPAIVs clade 2.3.4.4b from a fecal sample of falcated ducks (*Anas falcata*) and an environmental water sample collected in Japan in November 2020. 

The Izumi plain, which is in the Kagoshima Prefecture at the southern tip of Kyushu Island in Japan, is an overwintering site for several tens of thousands of wild migratory birds, including approximately 90% of the global population of the hooded crane (*Grus monachal*) and 50% of the white-naped crane (*Grus vipio*) [12,13]. Since these two cranes are classified as vulnerable species [14,15], various conservation measures, such as creating artificial wet paddies as roosts, have been conducted every winter. In addition to cranes, wild ducks, including mallards, northern pintails, and Eurasian wigeon, which are considered the primary natural reservoir of AIVs [11], also share this overwintering site [16]. In fact, both HPAIVs and low pathogenic AIVs were previously isolated from dead or debilitated cranes, duck feces, and water samples from the Izumi plain [5,17,18].

On 5 November 2020, duck fecal samples were collected by the local government authority at the Komenotsu River mouth, approximately 6 km away from a crane roost in the Arasaki area, during a public AIV surveillance. Influenza viral RNA was detected in one of the five pooled fecal samples using reverse transcription loop-mediated isothermal amplification (RT-LAMP) at the National Institute for Environmental Studies, as described previously [19]. We then inoculated the AIV gene-positive fecal specimen into embryonated chicken eggs for virus isolation, as described previously [16]. Using the rapid diagnostic test ESPLINE A Influenza (Fujirebio Inc., Tokyo, Japan), the allantoic fluids harvested from the inoculated eggs were found to test positive for influenza A viral antigen. To identify the duck species of the fecal sample, the cytochrome *c* oxidase I (COI) gene of the mitochondrial DNA was sequenced as described previously [20]. A Basic Local Alignment Search Tool (BLAST) search on the National Center for Biotechnology Information (NCBI) database revealed that the fecal sample was derived from falcated ducks. Subsequent genetic analyses of the allantoic fluid revealed that we isolated an AIV of the H5N8 subtype, named A/falcated duck/KU-d3/2020 (H5N8). Furthermore, on 9 November 2020, we collected environmental water samples from the crane roost in the Arasaki area during a private AIV surveillance and the water samples were subjected to AIV isolation in embryonated chicken eggs, as described previously [17]. We then isolated another H5N8 AIV, named A/environment/Kagoshima/KU-ngr-J2/2020 (H5N8). 

To genetically characterize the two H5N8 isolates, the nucleotide sequences of all eight gene segments from both isolates were determined (Table 1). The partial sequence of the HA genes showed a cleavage site motif of REKRRKR↓GLF, indicating the high pathogenicity in chickens. All eight gene segments from both isolates were almost identical to each other, with the nucleotide sequences of the HA and M genes from both isolates sharing 100% identity. These results suggest that feces from falcated ducks are a source of water contamination of crane roosts on the Izumi plain (Table 2).

To identify the closest relatives of all the viral gene segments from A/falcated duck/KU-d3/2020 (H5N8) isolate, the sequences were subjected to BLAST search against the Global Initiative on Sharing Avian Influenza Data (GISAID) and NCBI databases (Table 2). 

All eight gene segments from A/falcated duck/KU-d3/2020 (H5N8) showed high similarities (99.34–99.90%) to their counterparts from two H5N8 HPAIVs of clade 2.3.4.4b isolated in East Asia in October 2020, namely A/Mandarin duck/Korea/H242/2020 (H5N8) [9] and A/northern pintail/Hokkaido/M13/2020 (H5N8) [10]. Although the gene segments from A/falcated duck/KU-d3/2020 (H5N8) also showed high similarity to European poultry isolates from the winter of 2019/2020 [21], they were less similar than those against the Asian isolates. These findings indicated that H5N8 HPAIVs recently isolated in East Asia and Europe share a recent common ancestor without genetic reassortment. These results also confirm the critical role of migratory birds, including the falcated duck, in the dissemination of recent H5N8 HPAIVs. 

Falcated ducks, also known as falcated teals, are dabbling ducks that have a wide breeding range spanning eastern Siberia and Mongolia to northeastern China and northern Japan, with wintering grounds in southeast Asia to eastern India [22]. Hence, falcated ducks are considered one of the migratory ducks that may facilitate the dissemination of AIVs through their migratory flyways. In fact, falcated ducks, along with other wild birds, were implicated in the dissemination of H5N8 HPAIVs in East Asia during the outbreak in South Korea in January 2014 [3]. However, falcated ducks are a minor duck species that host AIVs: among 18,502 AIVs isolated from duck species deposited in the GISAID database as of 9 December 2020, only 0.037% were associated with falcated duck, whereas more AIVs were isolated from mallards (30.82%), northern pintails (4.79%), and Eurasian wigeons (0.28%). These three duck species can be also observed at the Izumi plain every winter as stated above. More importantly, A/falcated duck/KU-d3/2020 (H5N8) is the first falcated duck isolate in Japan. Our findings imply the potential of falcated ducks as one of the migratory ducks facilitating the dissemination of AIVs to Japan.

In conclusion, we isolated two H5N8 HPAIVs clade 2.3.4.4b from falcated duck feces and environmental water collected at an overwintering site for wild migratory birds in Japan in November 2020. Genetic analyses revealed that our isolates were almost identical, suggesting the potential of feces from falcated ducks as a source of environmental water contamination. In addition, the genomes of our isolates shared high similarity with those from H5N8 HPAIVs recently isolated in South Korea and the northern part of Japan, Hokkaido, implying the potential role of falcated ducks in the dissemination of HPAIVs. Our results highlight the importance of global surveillance of AIVs.

## Figures and Tables

**Table 1:** Accession number of each gene segment of the two characterized avian influenza viruses (AIVs).

Virus	Gene	Accession No.
A/falcated duck/KU-d3/2020 (H5N8)	PB2	MW342697 *
	PB1	MW342698
	PA	MW342699
	HA	MW342700
	NP	MW342701
	NA	MW342702
	M	MW342703
	NS	MW342704
A/environment/Kagoshima/KU-ngr-J2/2020(H5N8)	PB2	EPI1815131 **
	PB1	EPI1815132
	PA	EPI1815133
	HA	EPI1815134
	NP	EPI1815135
	NA	EPI1815136
	M	EPI1815137
	NS	EPI1815138

* Accession numbers in the GenBank database are listed. ** Accession numbers in the GISAID database are listed.

**Table 2:** Percent nucleotide identity of A/falcated duck/KU-d3/2020 (H5N8) with its closest relatives.

Gene	Accession No.*	Closest Relative **	Identity (%)
PB2	MW342697	A/environment/Kagoshima/KU-ngr-J2/2020 (H5N8)	99.52
		A/Mandarin duck/Korea/H242/2020 (H5N8)	99.42
		A/northern pintail/Hokkaido/M13/2020 (H5N8)	99.34
		A/domestic duck/Poland/285/2020 (H5N8)	99.16
PB1	MW342698	A/environment/Kagoshima/KU-ngr-J2/2020 (H5N8)	99.87
		A/Mandarin duck/Korea/H242/2020 (H5N8)	99.86
		A/northern pintail/Hokkaido/M13/2020 (H5N8)	99.78
		A/duck/Hungary/1565_20VIR749-2/2020 (H5N8)	99.42
PA	MW342699	A/environment/Kagoshima/KU-ngr-J2/2020 (H5N8)	99.91
		A/Mandarin duck/Korea/H242/2020 (H5N8)	99.81
		A/northern pintail/Hokkaido/M13/2020 (H5N8)	99.81
		A/domestic goose/Poland/028/2020 (H5N8)	99.39
HA	MW342700	A/environment/Kagoshima/KU-ngr-J2/2020 (H5N8)	100.0
		A/Mandarin duck/Korea/H242/2020 (H5N8)	99.76
		A/northern pintail/Hokkaido/M13/2020 (H5N8)	99.71
		A/chicken/Germany-BW/AI00049/2020 (H5N8)	99.53
NP	MW342701	A/environment/Kagoshima/KU-ngr-J2/2020 (H5N8)	99.87
		A/Mandarin duck/Korea/H242/2020 (H5N8)	99.93
		A/northern pintail/Hokkaido/M13/2020 (H5N8)	99.80
		A/chicken/Poland/003/2020 (H5N8)	99.53
NA	MW342702	A/environment/Kagoshima/KU-ngr-J2/2020 (H5N8)	99.93
		A/Mandarin duck/Korea/H242/2020 (H5N8)	99.57
		A/northern pintail/Hokkaido/M13/2020 (H5N8)	99.79
		A/turkey/Poland/096/2020 (H5N8)	99.07
M	MW342703	A/environment/Kagoshima/KU-ngr-J2/2020 (H5N8)	100.0
		A/Mandarin duck/Korea/H242/2020 (H5N8)	99.89
		A/northern pintail/Hokkaido/M13/2020 (H5N8)	99.90
		A/domestic duck/Poland/271/2020 (H5N8)	99.79
NS	MW342704	A/environment/Kagoshima/KU-ngr-J2/2020 (H5N8)	99.88
		A/Mandarin duck/Korea/H242/2020 (H5N8)	99.52
		A/northern pintail/Hokkaido/M13/2020 (H5N8)	99.52
		A/turkey/Czech Republic/3071/2020 (H5N8)	98.92

* Accession numbers in the GenBank database are listed. ** Top four viruses with the highest nucleotide identity found in the GISAID and/or GenBank databases on 2 December 2020 are listed. Note that the top three viruses for all gene segments are our isolate from the environmental water sample, followed by the recent South Korean isolate or the recent isolate from the northern part of Japan.

## Data Availability

The data that support the findings of this study are openly available in the GenBank at https://www.ncbi.nlm.nih.gov/genbank/, reference numbers MW342697-MW342704 and in the GISAID database at https://www.gisaid.org/, reference numbers EPI1815131-EPI1815138.
